# Imaging quality of an artificial intelligence denoising algorithm: validation in ^68^Ga PSMA-11 PET for patients with biochemical recurrence of prostate cancer

**DOI:** 10.1186/s13550-023-00999-y

**Published:** 2023-05-25

**Authors:** Charles Margail, Charles Merlin, Tommy Billoux, Maxence Wallaert, Hosameldin Otman, Nicolas Sas, Ioana Molnar, Florent Guillemin, Louis Boyer, Laurent Guy, Marion Tempier, Sophie Levesque, Alban Revy, Florent Cachin, Marion Chanchou

**Affiliations:** 1 Nuclear Medicine, CLCC Jean Perrin: Centre Jean Perrin, Clermont-Ferrand, France; 2 Inserm UMR 1240 IMOST, Physique Médicale, CLCC Jean Perrin, Université Clermont Auvergne, Clermont-Ferrand, France; 3 Incepto Medical, Paris, France; 4 Biostatistics, CLCC Jean Perrin, Clermont-Ferrand, France; 5 Inserm UMR1240 IMoST, Clermont-Ferrand, France; 6 Radiotherapy, CLCC Jean Perrin, Clermont-Ferrand, France; 7 Radiology, UMR 6602 UCA/CNRS/SIGMA, Hôpital Gabriel-Montpied TGI –Institut Pascal, Clermont-Ferrand, France; 8 Urology, Hôpital Gabriel-Montpied, Clermont-Ferrand, France; 9 Université Clermont Auvergne, Clermont-Ferrand, France

**Keywords:** ^68^Ga PSMA-11, PET, Artificial intelligence algorithm, Biochemical recurrence of prostate cancer, Image quality, Denoising

## Abstract

**Background:**

^68^ Ga-PSMA PET is the leading prostate cancer imaging technique, but the image quality remains noisy and could be further improved using an artificial intelligence-based denoising algorithm. To address this issue, we analyzed the overall quality of reprocessed images compared to standard reconstructions. We also analyzed the diagnostic performances of the different sequences and the impact of the algorithm on lesion intensity and background measures.

**Methods:**

We retrospectively included 30 patients with biochemical recurrence of prostate cancer who had undergone ^68^ Ga-PSMA-11 PET-CT. We simulated images produced using only a quarter, half, three-quarters, or all of the acquired data material reprocessed using the SubtlePET® denoising algorithm. Three physicians with different levels of experience blindly analyzed every sequence and then used a 5-level Likert scale to assess the series. The binary criterion of lesion detectability was compared between series. We also compared lesion SUV, background uptake, and diagnostic performances of the series (sensitivity, specificity, accuracy).

**Results:**

VPFX-derived series were classified differently but better than standard reconstructions (*p* < 0.001) using half the data. Q.Clear series were not classified differently using half the signal. Some series were noisy but had no significant effect on lesion detectability (*p* > 0.05). The SubtlePET® algorithm significantly decreased lesion SUV (*p* < 0.005) and increased liver background (*p* < 0.005) and had no substantial effect on the diagnostic performance of each reader.

**Conclusion:**

We show that the SubtlePET® can be used for ^68^ Ga-PSMA scans using half the signal with similar image quality to Q.Clear series and superior quality to VPFX series. However, it significantly modifies quantitative measurements and should not be used for comparative examinations if standard algorithm is applied during follow-up.

## Introduction

Artificial intelligence (AI) is finding its way into medicine through medical imaging. AI algorithms can be used at two different points in the processing of cross-sectional images, i.e., either during or after iterative image reconstruction [1]. The SubtlePET® algorithm (SubtlePET, Incepto Medical) is a deep learning-based post-reconstruction image denoising algorithm [2] used in PET imaging. The algorithm is a supervised convolutional neuronal network (CNN) based on a 2.5D encoder–decoder with a U-net shape. It has not been trained in our center [3] but has been trained on millions of images through dataset augmentation [4]. The version 1.3 of the algorithm we used in this study was trained only on FDG and Amyloid PET (PET/CT and PET/MRI). No specific hardware is needed to use this algorithm, and the time between the end of examination and the reconstruction is shorter than 10 min. SubtlePET® is a denoising algorithm that focuses on making the images smoother, and not a deblurring algorithm that would sharpen the lesion contours. This algorithm is already validated by the Food and Drug Administration for tracers marked with Fluorine-18 (^18^FDG [3] and Amyloid PET [5]) and can divide acquisition time by four without losing image quality [3]. It has not been investigated or trained for different isotope such as the gallium-68 encountered in prostate oncology imaging.

Prostate cancer is the most common cancer in men, and the third leading cause of cancer-related mortality [6]. It has a very high relapse rate of about 50% [7], which is explained by a lack of sensitivity of conventional imaging techniques [8]. Prostate cancer does not show particularly high glucose uptake, and this has prompted the development of dedicated tracers, i.e., prostate-specific membrane antigen (PSMA) ligands, which are typically labeled with gallium-68 in current practice. PSMA is predominantly expressed by prostate cells, and its binding intensity increases with aggressiveness of the cancer [9]. PET with PSMA ligands has shown high diagnostic performances in both initial staging [10] and biological recurrence [11–13]. Furthermore, gallium-68 has to be produced by a generator, which significantly limits the use of ^68^ Ga-PSMA in current practice [14].

The wider context of growing public health issues and the development of targeted radiotherapy (TRT) are driving a strong increase in demand for examinations. Reducing acquisition time while maintaining optimal or sufficient image quality would help to overcome the challenge of meeting the increasing needs without penalizing performance. AI, and possibly the SubtlePET® algorithm, could provide solutions by maintaining a satisfactory image quality despite a reduced count statistic. This study applies transfer learning [1], as the SubtlePET® algorithm has not yet been tested with gallium-68-labeled tracers.

This study set out to assess the influence of PET images obtained using the SubtlePET® algorithm on ^68^ Ga-PSMA PET image quality under four levels of information loss compared to images obtained by standard reconstruction. The secondary objective was to analyze the diagnostic performances of the different PET series (post-processed or not by SubtlePET®) and the influence of SubtlePET® on lesion intensity and background measures.

## Methods

### Patients

We retrospectively included 30 consecutive patients aged over 18 years old presenting with biochemical recurrence of prostate cancer at the Jean Perrin Cancer Center (Clermont-Ferrand, France) for ^68^ Ga-PSMA PET from July 17 to October 20, 2020. As per French regulations, every patient had already undergone ^18^F-choline PET/CT that were negative or inconclusive [15]. The data collected was age, weight, Gleason score, ISUP score, prostate cancer treatment, initial prostate-specific antigen (PSA) level, and last known PSA level before the PET scan.

Every patient received an information letter validated by the Jean Perrin Cancer Center data protection department and was free to refuse use of their data throughout the study.

### Image acquisition

The tracer used is ^68^Ga PSMA-11, also called HBED-CC, Glu-urea-Lys(Ahx)-HBED-CC, or PSMA-HBED-CC [8]. Image acquisition usually begins 60 min after IV injection of ^68^ Ga-PSMA-11 with an average activity of 2.0 MBq/kg [16] (118–453 MBq) with 4 min per scan step from the vertex to the upper third of the femur. Excluding one patient, the range of activity is thinner (118–224 MBq). A low-dose CT scan was performed for attenuation correction and localization.

Fifteen scans were performed on a PMT-based Discovery 710 Optima 660® scanner, and 15 were performed on a SiPM-based Discovery MIDR® scanner (GE Health, Healthcare, Milwaukee, WI). No cross-validation was made across the two scanners. Both machines have a 700-mm field of view, 256 × 256 matrix, and 2.7 × 2.7 × 3.27 mm^3^ voxel volumes. The CT parameters of the SiPM-based PET were 124 kV of mean tube voltage (range 120–140 kV), 76.2 mA of mean tube current (range 58–159 mA), a pitch of 0,98, a noise index of 28,2, and a percentage of iterative reconstruction of 40%. The CT parameters of the PMT-based PET were 128 kV of mean tube voltage (range 120–140 kV), 76.2 mA of mean tube current (range 55–123 mA), a pitch of 0.98, a noise index of 27.3, and a percentage of iterative reconstruction of 40%. No cross-validation was done across the two scanners. For each scan, two standard images were reconstructed, the first with VPFX (ordered subset expectation maximization (OSEM) + time of flight (TOF)) and the second with Q.Clear (OSEM + TOF + point spread function (PSF)) [17]. We named these standard reconstructions VP4 and QC4, respectively. The VPFX series were performed with two iterations for 24 subsets.

The algorithm uses a residual learning approach optimized for quantitative (L1 norm) as well as structural similarity. It learns to separate and suppress the noise components while preserving and enhancing the structural components. The networks were trained with paired low- and high-count PET series coming from a wide range of clinical indications and patient BMIs and from a large variety of PET/CT and PET/MR devices (10 General Electric, 5 Siemens, and 2 Philips models). The training data included millions of paired image patches derived from hundreds of patient scans with multi-slice PET data and data augmentation. The list mode allows retrospective reconstruction of images by artificially reducing the count statistic by taking only the data acquired in a given time-interval. For each patient, we used on 1.3.0 of the SubtlePET® post-processing denoising algorithm to create 4 image series equivalent to one-minute, two-minute, three-minute and four-minute steps. We named these series SVP1, SVP2, SVP3, and SVP4 for the VPFX-derived series and SQC1, SQC2, SQC3, and SQC4 for the Q.Clear-derived series, respectively. These series were anonymized by a medical physicist blind to the image interpreting team.

### Image analysis

Three nuclear physicians blindly interpreted every series using the PET VCAR module bundled with the General Electrics’ ADW® software. These 3 readers had different degrees of experience: One was a PSMA referent (16-year experience), one was a senior physician (4-year experience), and one was a resident nuclear physician (1-year experience). All three readers had experience reading PSMA PET scans in their daily practice. The VP4 and QC4 series analyzed by the most experienced reader served as the gold standard benchmark for assessing the other series.

Data for lesions suspected of malignancy were collected by classifying the lesions according to anatomical location, i.e., prostate bed, pelvic lymph nodes, secondary bone lesions, or secondary extra-osseous. In the event of lymph node or bone involvement, the precise locations were recorded to facilitate comparison between series. Lesions were classified into 5 levels [18]: negative, equivocal negative, equivocal, equivocal positive, or positive. Lesions labeled as equivocal positive or positive were considered significant.

### Image quality

We evaluated the quality of each series based on 3 criteria: overall image quality, interpretability, and visualization or not of suspected lesions. For overall image quality, we used a 5-point Likert scale [5, 19] for noise level, contrast, and signal-to-noise ratio estimated visually (1 = uninterpretable, 2 = bad, 3 = correct, 4 = good, 5 = excellent). We retained levels 3, 4, and 5 as usable in daily practice, thus classifying the series as interpretable.

We also evaluated image quality using two binary indexes: series interpretability and lesion detectability.

### Quantitative analysis

Quantitative analyses focused on SUVmean and SUVmax measurements, where SUV is ‘standardized uptake value.’ SUVmean measures the mean activity in a volume, whereas SUVmax retains only the value of the hottest pixel. To evaluate the influence of the algorithm on SUV measurements, we recorded the SUVmax of every lesion found on each examination and background measurement performed.

SUVmax was measured by plotting regions of interest on each lesion by semi-automatic method. For homogenization purposes, the analyses for each specific anatomical location only considered the most intense lesions of each patient. SubtlePET®-induced bias in lesion SUVmax measurements was evaluated using the following formula: (study series SUVmax—reference series SUVmax)/reference series SUVmax [1].

Background measurements were performed by the junior reader using a 2-cm-diameter sphere in the right liver and right gluteal muscle and a 1-cm-diameter circle in the aortic arch [20, 21]. We chose to take the gluteal region as background reference as most lesions in this specific application of prostate pathology are located in the pelvic area. The SUVmean and SUVmax of the background uptake in these regions of interest were measured. Signal-to-background ratio (SBR) was defined by the following formula: lesion SUVmax/background uptake SUVmean [22]. We also performed subgroup analysis based on PSA level, weight, and camera type.

#### Diagnostic performance of PET series using the SubtlePET® denoising algorithm

To evaluate the diagnostic performance of the SubtlePET® series, we performed a series-by-series analysis per reader. Our gold standard benchmarks were our most experienced reader and the routine-process reconstruction series, i.e., not reprocessed by the denoising algorithm.

True positives were defined as lesions classified as positive by the most experienced reader in the usual reconstruction series (QC4 and VP4) as well as by all readers in all other series. True negatives were defined as lesions classified as negative by the most experienced reader in the usual series and negative by all readers in all other series. False positives were defined as lesions classified as negative or not found by the most experienced reader in the usual series but classified as positive in the other series. False negatives were defined as lesions classified as positive by the most experienced reader in the usual reconstruction series but classified as negative in the other series.

We were thus able to calculate the sensitivity, specificity, and accuracy of each of the series processed by SubtlePET® for each reader.

### Statistical analysis

To lend clarity to the statistical analysis, we pooled lesions classified as equivocal positive into the positive group and lesions classified as equivocal and equivocal negative into the negative group.

Subgroup analyses were stratified by patient weight, PSA values, and the two different cameras.

Analyses involving image quality (categorical variables) were compared using Cochran’s Q test for differences between all readers and McNemar’s test for pairwise comparison.

Analyses involving quantitative parameters (continuous variables) were compared by the Student’s paired-samples *t* test and the Wilcoxon signed rank test.

A p value adjustment was performed́ to account for the multiplicity of tests. Statistical analyses were performed with R software version 4.1.0 (R-Project, GNU GPL, http://cran.r-project.org/).

## Results

### Population

The characteristics of the patient population are summarized in Tables 1 and 2. Our patients had a mean age of 67.6 years, a majority of Gleason 7 prostate cancer (80%), and a median PSA level before PET of 0.682 ng/mL (min 0.23 ng/mL–max 20 ng/mL). Most patients had been initially treated with radical prostatectomy (89.6%). One patient had prostate cancer at initial diagnosis but was not biochemically recurrent. Average injected activity was 2.09 MBq/kg (min 1.51 MBq/kg–max 5.03 MBq/kg) in accordance with European recommendations [16]. One of the patients was injected with higher-than-normal activity, but there was no recorded evidence of toxicity.

**Table 1:** Characteristics of the patient population

	Median	Mean	Min	Max
Age years	67	67.56	55	81
Weight kg	84	83.4	63	116
Injected activity MBq	164	174.1	118	453
Activity/kg MBq/kg	1.97	2.09	1.51	5.03
Initial PSA ng/mL	11	10.7	5.7	17.46
Last PSA before PET ng/mL	0.682	2.356	0.23	20

**Table 2:** Characteristics of the patients at diagnosis

			n
Initial Gleason score	6		1
	7		24
		*3* + *4*	*12*
		*4* + *3*	*12*
	8		3
	9		1
	Not available		1
Initial ISUP score	1		1
	2		12
	3		12
	4		3
	5		1
	Not available		1
Initial treatment	Prostatectomy		26
	Radiotherapy		3
	None		1

### Number and localization of lesions

**Fig. 1:**
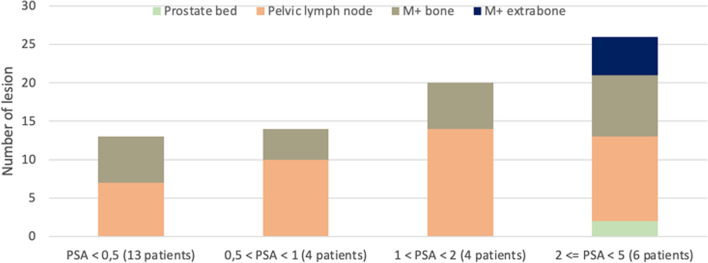
Location of lesions according to PSA level for the gold standard series interpreted by the referent reader. The column with patients whose PSA level was higher than 5 has been omitted, because after excluding patients at initial staging, there was only one patient with lesions left in this subgroup, making it insignificant

**Fig. 2:**
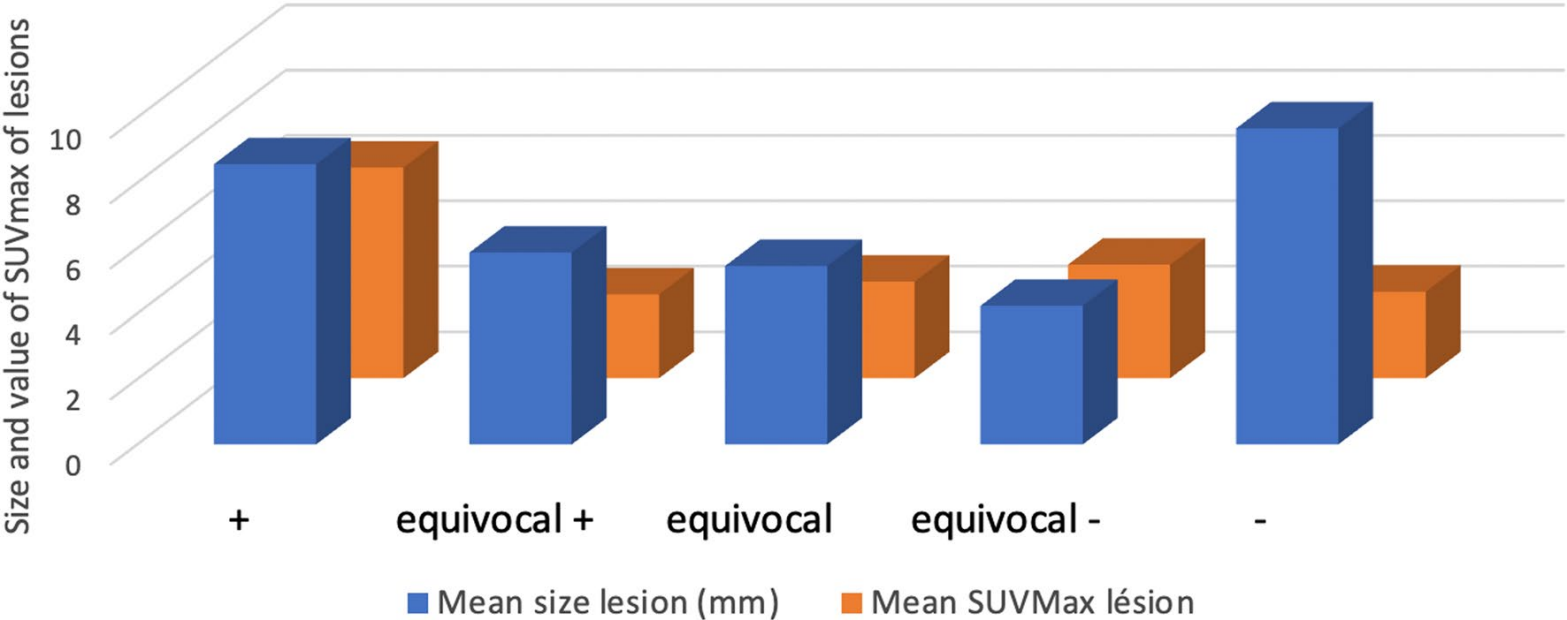
Mean size and mean SUVmax of all lesions

A total of 1919 lesions were found, i.e., an average of 2.13 lesions per series, of which 1329 (i.e., 69.25%) were clinically significant. Suspicious lesions were found in 25 out of 30 patients. The referent reader found 658 lesions, of which 378 were significant. The senior reader found 574 lesions, of which 425 were significant. The junior reader found 687 lesions, of which 526 were significant. The total number of lesions per series is reported in Table 3. Among the significant lesions, 121 were localized in the prostatic bed, 717 in the lymph nodes, 428 in the bones, and 63 in the extra-osseous tissues. Figure 1 plots the number and location of the lesions according to PSA levels. Mean size was 8.6 mm for positive lesions and 5.9 mm for equivocal-positive lesions. Figure 2 plots mean size of lesions compared to mean SUVmax. Readers tended (*p* > 0.062) to classify lesions more significantly on the gold standard series.

**Table 3:** Number and classification of lesions stratified by series and reader

	QC4	SQC1	SQC2	SQC3	SQC4	VP4	SVP1	SVP2	SVP3	SVP4	Total
REFERENT	**67**	**62**	**62**	**64**	**67**	**71**	**65**	**63**	**69**	**68**	**658**
−	9	11	13	11	9	10	9	12	8	8	100
Equivocal −	11	9	5	9	13	13	12	6	16	13	107
Equivocal	6	8	7	7	8	6	7	6	8	10	73
Equivocal +	9	4	6	6	6	11	6	7	6	6	67
+	32	30	31	31	31	31	31	32	31	31	311
SENIOR	**57**	**58**	**57**	**58**	**57**	**57**	**59**	**57**	**57**	**57**	**574**
−	1	4	2	2	1	1	4	2	1	1	19
Equivocal −	12	10	11	15	15	10	12	10	12	13	120
Equivocal	1	1	1	1	1	1	1	1	1	1	10
Equivocal +	7	7	7	5	5	6	5	7	8	6	63
+	36	36	36	35	35	39	37	37	35	36	362
JUNIOR	**68**	**68**	**69**	**68**	**68**	**72**	**69**	**70**	**68**	**67**	**687**
−	6	7	7	7	7	5	8	8	6	6	67
Equivocal −	5	4	10	3	6	2	6	4	3	1	44
Equivocal	4	2	4	4	5	3	6	6	7	9	50
Equivocal +	13	14	11	14	13	12	11	12	12	12	124
+	40	41	37	40	37	50	38	40	40	39	402
Total	192	188	188	190	192	200	193	190	194	192	1919

### Image quality analysis

#### Overall image quality and interpretability

#### VPFX

We a found a significant difference (*p* < 0.001) in overall quality of the VP4, SVP1, SVP2, SVP3, and SVP4 series, with, respectively, 65.6%, 36.6%, 85%, 91.1%, and 100% of the series classified as correct or over. The junior reader had more misclassified (*p* < 0.05) VPFX, SVP2 and SVP3 series compared to the other readers. The senior reader classified the SVP1 series better compared to the other readers (*p* < 0.001).

Almost all (99.6%) SVP2, SVP3, and SVP4 series were classified as interpretable by all readers, and 65.6% of the SVP1 series and 83.3% of the VP4 series were classified as interpretable by all readers. Thus, in the overall comparison between series, the series processed by SVP2, SVP3, and SVP4 were significantly (*p* = 0.001) better classified than the gold standard series (VPFX).

### Q.Clear

The only statistically significant difference (*p* < 0.001) found was between the QC4 and SQC1 series, with 91.1% and 42.2% of the series classified as correct or better, respectively. There was no statistically significant difference in quality between the QC4 series, and the SQC2, SQC3, and SQC4 series, of which 93.3%, 93.3%, and 94.4%, respectively, were rated correct or better.

100% of the QC4, SQC4, SQC3, and SQC2 series were interpretable by all readers versus 64.4% of the SQC1 series.

### Lesion detectability

**Fig. 3:**
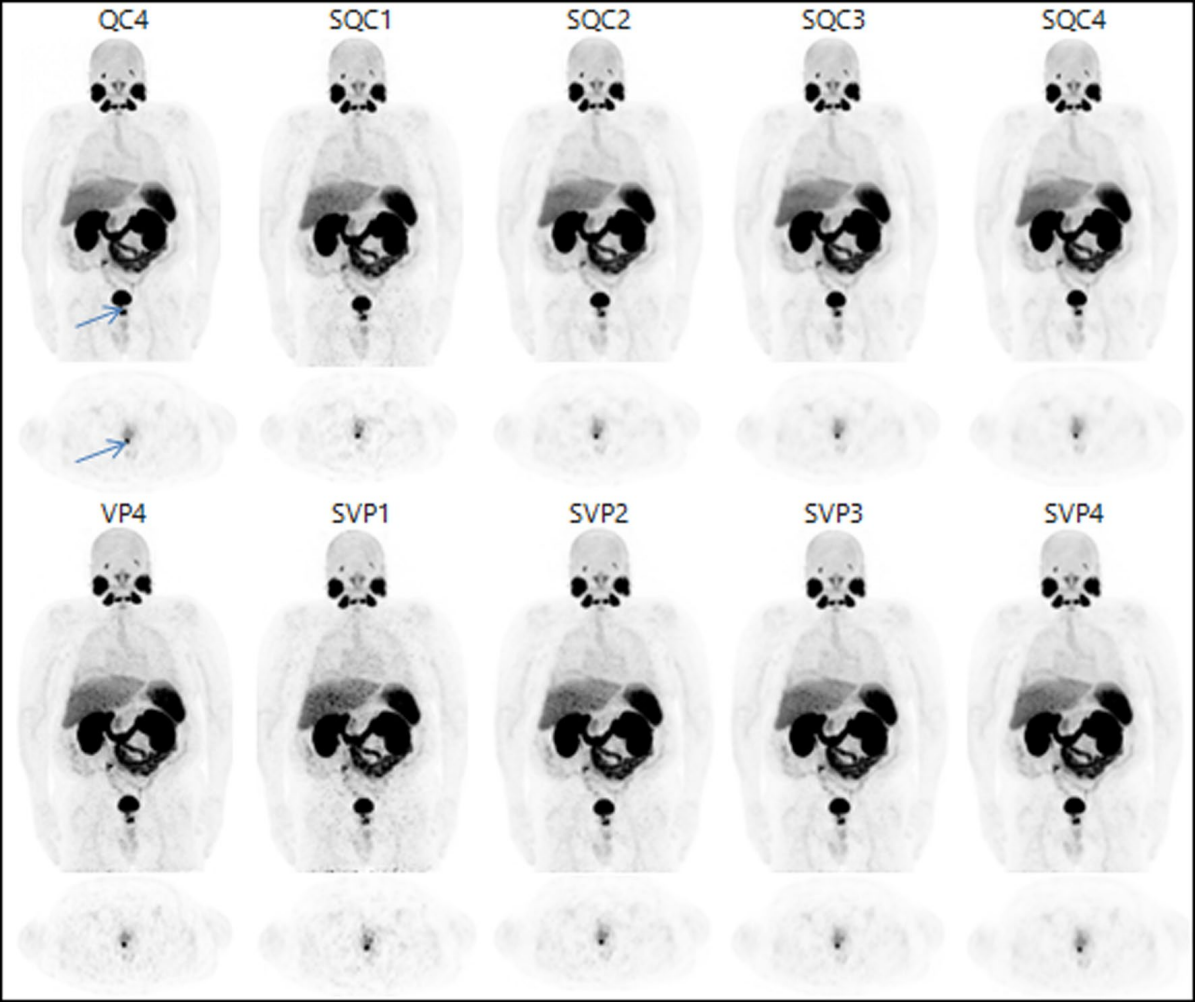
Patient 1: 112 kg, 1.83 MBq/kg, PSA before PET 20 ng/mL. 3D MIP images on the first lines and axial section on the 2nd lines. The arrow shows a prostatic bed lesion. LUT range 0–10 g/mL

**Fig. 4:**
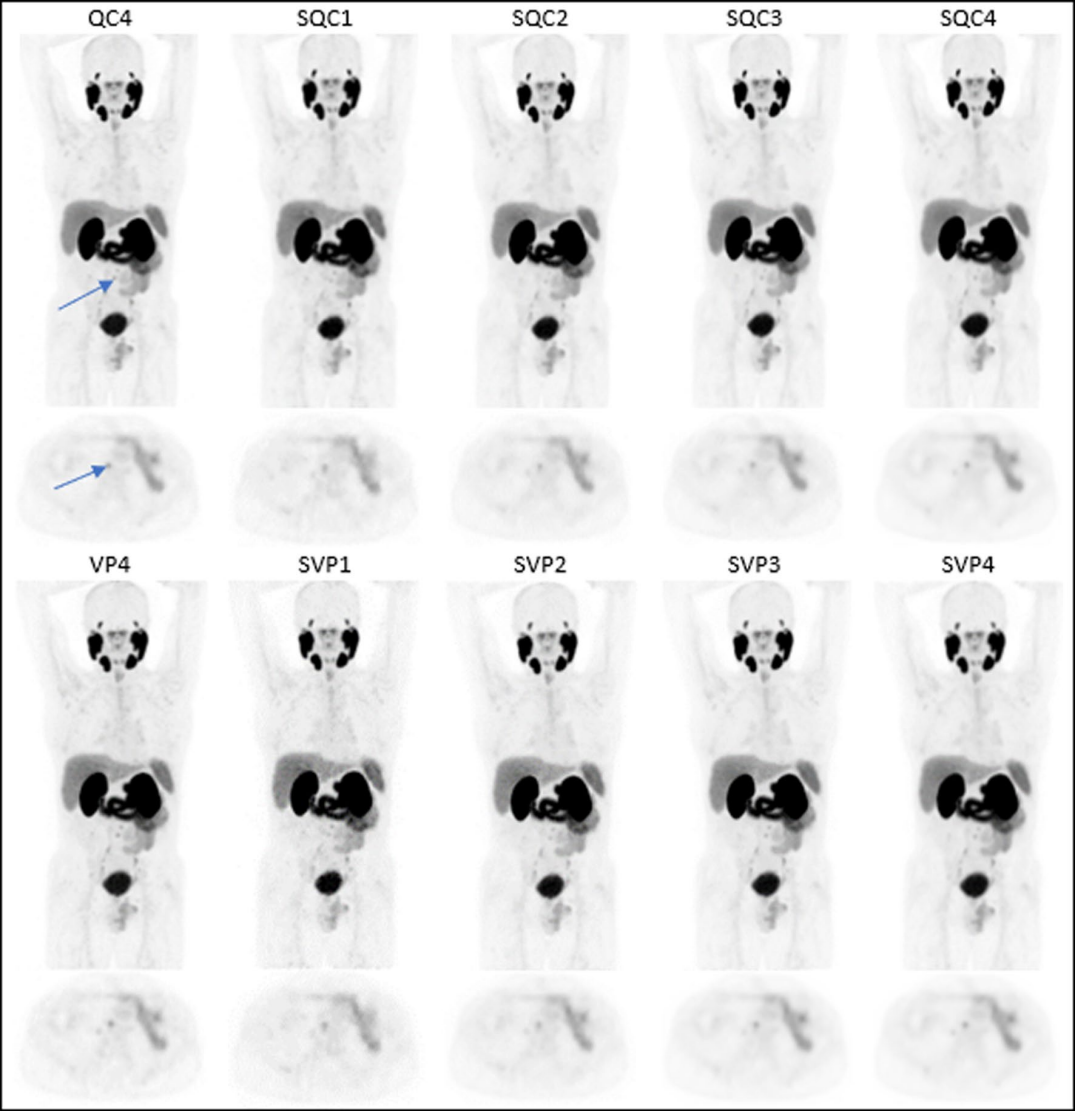
Patient 2. 63 kg, 3.03 MBq/kg, PSA before PET 2 ng/mL. 3D MIP images on the first lines and axial section on the 2nd lines. The arrow shows a retroperitoneal lymph node. LUT range 0–10 g/mL

Concerning lesion visualization, there was a statistically significant difference between readers (*p* < 0.001) but not in the between-series analysis. Note that even though some series were classified as uninterpretable, lesion detection was still possible. Figures 3 and 4 give illustrative examples of the image quality achieved (Fig. 4).

### Analysis of quantitative parameters

#### Influence of the denoising algorithm on lesion SUVmax

The mean SUVmax value was 8.91 for QC4 and 7.7 for VP4. There was a statistically significant decrease (*p* < 0.05) in measured SUVmax values of lesions in all the series reconstructed with SubtlePET® except for SQC1. The less data acquired in the series, the closer the SUVmax values were to the reference value. The results are reported in Table 4. Further analysis of lesion SUVmax values stratified by lesion size revealed that our cohort only had small lesions, which are particularly sensitive to the partial volume effect (see Fig. 2).

**Table 4:** Differences in SUVmax of lesions between the series treated with SubtlePET® and the standard series

Series	Mean of SUVmax lesion	Difference SUVmax lesion	CI 95% of Difference of SUVmax lesion	*p* adjusted	Bias (%)
QC4	8.91				
SQC1	8.30	0.49	(− 0.27–1.31)	0.187	− 5
SQC2	7.52	0.96	(0.45–1.58)	*0.002*	− 15
SQC3	7.61	1.17	(0.74–1.72)	< *0.001*	− 16
SQC4	7.41	1.41	(0.93–1.88)	< *0.001*	− 18
VP4	7.70				
SVP1	6.67	0.97	(0.11–1.91)	*0.036*	− 14
SVP2	6.82	0.95	(0.54–1.78)	*0.006*	− 16
SVP3	6.67	1.08	(0.6–1.75)	*0.006*	− 18
SVP4	6.72	1.18	(0.83–1.56)	*0.001*	− 17

Difference = Gold standard—study series; Bias = (Study series—Gold standard)/Gold standard

### Influence of the denoising algorithm on background uptake SUV (Appendix 1)

For VPFX-derived series, mean SUVmean was 1.34 for the aorta, 5.03 for the liver, and 0.39 for the right gluteal muscle. Vascular background was significantly higher (p < 0.05) for all series using only half the signal. Liver background was significantly higher (*p* = 0.001) for all series treated with SubtlePET®. Gluteal background was significantly lower (*p* < 0.05) for all series processed with SubtlePET®. The decrease was only a modest 0.04 at most, which is not clinically significant but could still facilitate the detection of low-intensity lesions, especially in this indication.

For Q.Clear-derived series, mean SUVmean was 1.09 for the aorta, 5.07 for the liver, and 0.39 for the right gluteal muscle. Liver and gluteal background were significantly different (*p* < 0.05), with a mean increase of 0.23 in liver values and a mean decrease of 0.03 in gluteal values. Other quantitative parameters were not significant.

Results of background SUVmax relative to VPFX and Q.Clear series are shown in Appendix 1.

### *Influence of the denoising algorithm on signal-to-background ratio (*Table 5*)*

**Table 5:** Comparison of the signal-to-background ratios (SBR)

Series	Mean	Difference SBR	CI 95% of difference	*p* adjusted	Bias (%)
*Signal/Background = SUVmax lesion/SUVmean aorta*					
QC4	5.28				
SQC1	4.87	− 0.52	(− 1.22–0.3)	0.183	− 7.8
SQC2	4.37	− 0.94	(− 1.51–0.3)	*0.009*	− 17.2
SQC3	4.37	− 1.11	(− 1.58–0.62)	*0.001*	− 17.2
SQC4	4.28	− 1.16	(− 1.64–0.74)	< *0.001*	− 18.9
VP4	5.19				
SVP1	4.21	− 1.08	(− 2.22–0.07)	0.054	− 18.9
SVP2	4.11	− 1.29	(− 2.09–0.58)	*0.007*	− 20.8
SVP3	4.02	− 1.66	(− 2.13–0.56)	*0.006*	− 22.5
SVP4	4.17	− 1.27	(− 2.47–0.8)	*0.002*	− 19.6
*Signal/Background = SUVmax lesion/SUVmean liver*					
QC4	1.13				
SQC1	1.04	− 0.14	(− 0.29–0)	0.061	− 7.9
SQC2	0.92	− 0.19	(− 0.29–0.08)	*0.002*	− 18.5
SQC3	0.92	− 0.22	(− 0.36–0.14)	< *0.001*	− 18.5
SQC4	0.88	− 0.29	(− 0.42–0.19)	< *0.001*	− 22.1
VP4	1.07				
SVP1	0.89	− 0.25	(− 0.52–0.05)	*0.02*	− 16.8
SVP2	0.85	− 0.22	(− 0.35–0.1)	*0.007*	− 20.6
SVP3	0.84	− 0.25	(− 0.47–0.16)	*0.004*	− 21.5
SVP4	0.85	− 0.28	(− 0.44–0.2)	*0.001*	− 20.6
*Signal/Background = SUVmax lesion/SUVmean gluteal*					
QC4	14.89				
SQC1	15.36	0.80	(2.06–2.94)	0.456	3.2
SQC2	13.60	− 1.01	(− 2.74–1.12)	0.247	− 8.7
SQC3	13.60	− 1.37	(− 2.34–0.2)	0.42	− 8.7
SQC4	13.29	− 2.07	(− 3.22–0.85)	0.13	− 10.7
VP4	13.96				
SVP1	12.53	− 1.81	(− 3.58–0.92)	0.267	− 10.2
SVP2	12.91	− 0.88	(− 2.51–0.91)	0.214	− 7.5
SVP3	12.63	− 1.66	(− 2.65–0.36)	0.084	− 9.5
SVP4	12.67	− 1.76	(− 2.47–0.8)	0.17	− 9.2

Mean SBR with the gluteal reference was 14.89 for QC4 and 13.89 for VP4. No significant difference was found for the SBR with the right gluteal region as reference. This result confirms the stability of lesion detectability even if the series were not considered visually interpretable.

Taking the hepatic background as reference, a statistically significant difference (*p* < 0.05) was demonstrated for all series except for SQC1. We thus showed a very modest decrease in the signal-to-background ratio (between − 0.28 and − 0.14 depending on the series).

There was a modest but significant (*p* < 0.05) decrease in signal-to-background ratios with the vascular region as the reference for the series computed from half the signal.

### Subgroup analysis

#### Subgroup analysis by PSA on PET scan

**Fig. 5:**
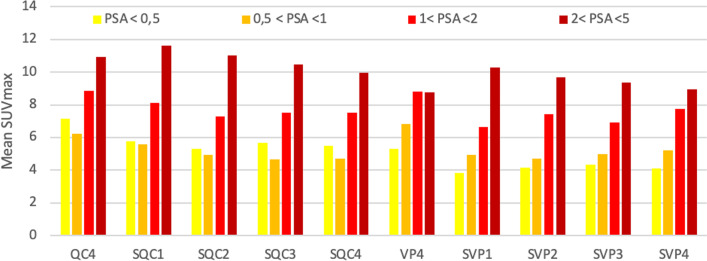
Mean SUVmax of lesions per series as a function of PSA

In the subgroup analysis on PSA on PET, we excluded the patient at initial staging. This left only one patient in the subgroup with a PSA level above 5 ng/mL, and so we did not perform analysis on this subgroup. The results are illustrated in Fig. 5. SUVmax values increased with PSA level except for patients with a PSA level lower than 1.

#### Subgroup analysis by weight

Despite our small number of patients, we performed a subgroup analysis by weight. The difference was only statistically significant (*p* < 0.05) for patients with a weight higher than median in SVP2, SVP3, SVP4, SQC2, SQC3, SQC4 and in patients lower than median SQC3, SQC4. Results were expressed with the definition of the signal-to-background ratio as (SUVmax lesion/SUVmean liver) and reported in Appendix 2. SubtlePET® tended to lower the signal-to-background ratios more often within heavier patients in the VPFX-derived series.

#### Subgroup analysis by camera

We performed a subgroup analysis to see the impact of the camera on the level of background in each series. There was a slight decrease in SUVmean that was only statistically significant in the gluteal region and the aortic region for VP4 and the Q.Clear-derived series on the newest camera. Results are shown in Appendix 3. Furthermore, there was no statistically significant difference with in the le level of lesion SUVmax or in SBR.

### Diagnostic performance (Table 6, Figs. 6 and 7)

**Table 6:** Diagnostic performance

Series	Reader	Accuracy (CI 95%)	Adjusted *p*	Cohen’s Kappa	Sensitivity	Specificity
VP4	Referent	1		1	1	1
	Senior	0.65 (0.48–0.79)	0.076	0.28	0.86	0.42
	Junior	0.77 (0.64–0.87)	< *0.001*	0.55	0.97	0.59
SVP1	Referent	0.98 (0.9–1)	< *0.001*	0.96	0.97	1
	Senior	0.70 (0.53–0.83)	*0.019*	0.39	0.86	0.53
	Junior	0.91 (0.8–0.97)	< *0.001*	0.82	0.93	0.88
SVP2	Referent	0.98 (0.9–1)	< *0.001*	0.97	1	0.97
	Senior	0.7 (0.53–0.83)	*0.019*	0.39	0.86	0.53
	Junior	0.81 (0.68–0.9)	< *0.001*	0.62	0.86	0.78
SVP3	Referent	1 (0.94–1)	< *0.001*	1	1	1
	Senior	0.68 (0.5–0.81)	*0.04*	0.34	0.81	0.53
	Junior	0.77 (0.64–0.87)	< *0.001*	0.54	0.83	0.71
SVP4	Referent	1 (0.94–1)	< *0.001*	1	1	1
	Senior	0.72 (0.56–0.85)	*0.008*	0.44	0.86	0.58
	Junior	0.81 (0.68–0.9)	< *0,001*	0,62	0,9	0,72
QC4	Referent	1		1	1	1
	Senior	0.75 (0.58–0.87)	*0.007*	0.48	0.86	0.61
	Junior	0.91 (0.8–0.97)	< *0.001*	0.82	0.93	0.88
SQC1	Referent	0.98 (0.9–1)	< *0.001*	0.96	0.97	1
	Senior	0.72 (0.56–0.85)	*0.018*	0.43	0.86	0.56
	Junior	0.84 (0.71–0.92)	< *0.001*	0.67	0.9	0.77
SQC2	Referent	0.98 (0.9–1)	< *0.001*	0.96	0.97	1
	Senior	0.75 (0.58–0.87)	*0.007*	0.48	0.86	0.61
	Junior	0.89 (0.78–0.96)	< *0.001*	0.78	0.93	0.85
SQC3	Referent	0.98 (0.9–1)	< *0.001*	0.97	0.97	1
	Senior	0.75 (0.58–0.87)	*0.007*	0.48	0.86	0.61
	Junior	0.89 (0.77–0.96)	< *0.001*	0.78	0.93	0.85
SQC4	Referent	0.98 (0.91–1)	< *0.001*	0.97	0.97	1
	Senior	0.75 (0.58–0.87)	*0.007*	0.48	0.86	0.61
	Junior	0.88 (0.75–0.95)	< *0.001*	0.75	0.9	0.85

**Fig. 6:**
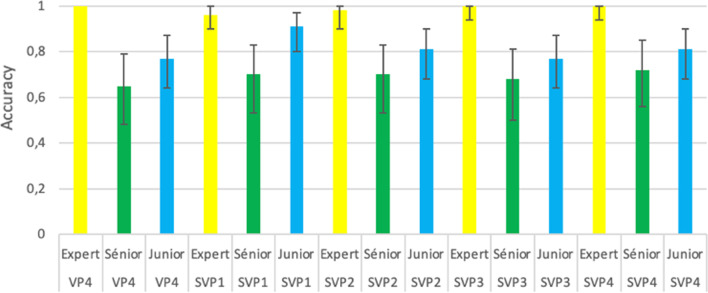
Accuracy (95%CI) for the VPFX series

**Fig. 7:**
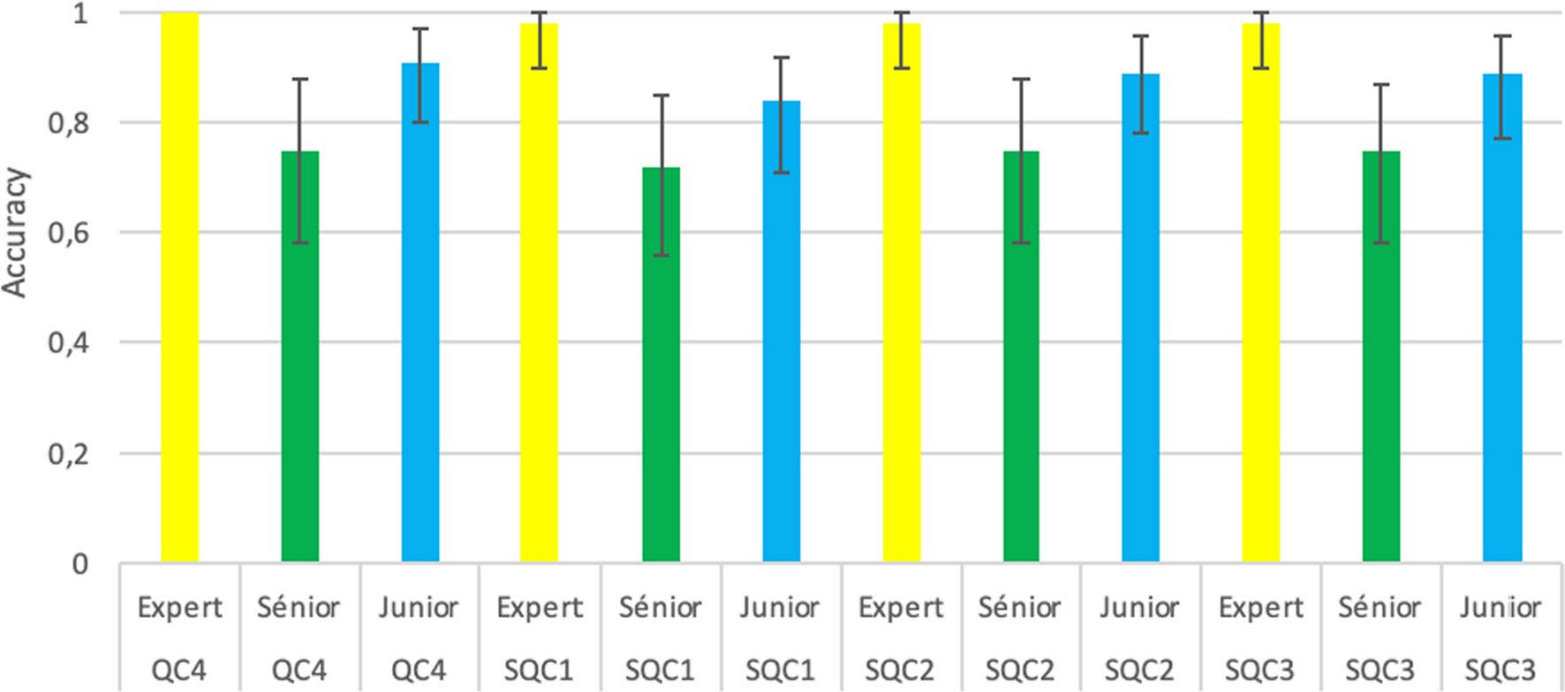
Accuracy (95%CI) for the Q.Clear series

Diagnosis accuracy was statistically compared, respectively, between algorithm models and readers. Few differences were shown between images examined by the same reader. Accuracy was superior to 0.98 whatever the algorithm used by the referent. However, analysis was statistically different between senor or junior readers and referent.

## Discussion

The SubtlePET® algorithm significantly improved (*p* < 0.001) the quality of VPFX-derived images computed from half the data (*p* < 0.001). For Q.Clear series, there was no statistically significant difference between series using at least half of the signal, knowing that Q.Clear already optimizes the images. Note that this analysis of image quality remains subjective. Even if some series were of insufficient quality, lesion detectability did not differ (*p* < 0.27) for all series. Regarding the influence of SubtlePET® on SUV measurements, the algorithm significantly lowered the lesion values (*p* < 0.005). The liver background measured by SUVmean increased significantly in both types of reconstruction, but this change was not clinically significant. Background SUVmax measurements followed a more contrasted pattern, with an increase in the Q.Clear-derived series but a decrease in the SVP4 series. Moreover, the SUVmax measurement of background regions was not very relevant because not including in the SBR. There was a slight decrease in the SUVmean of the aorta or gluteal on the PMT-based PET scan. Even if this decrease was light (< 0.18 g/mL), it might change the significant of small lesions when interpreting according to the PROMISE criteria [23]. The algorithm did not influence the diagnostic performance of each reader.

### Strengths

We were able to establish external validation of using the algorithm, as it was not trained or supervised at our center [3]. Moreover, as the SubtlePET® algorithm is a post-reconstruction image processing tool, it is independent of machine brand or model and therefore allows flexibility. In addition, two recent French studies [22, 24] found similar results on the main objective of image quality with only half the activity injected. One of these studies [22] used the Philips technology. Our results on SBR, lesion intensity, and level of background activity were also concordant even though we used a different radiopharmaceutical tracer.

The differences in image quality achieved for the VPFX series were very encouraging. For the series derived from the Q.Clear algorithm, which has already been optimized, our study suggests an equivalent quality of images produced when using only half of the data.

Signal-to-noise ratio correlates with detectability [18, 25]. We thus find a correlation in our study between analysis and detectability which remains stable despite a decrease in image quality. This improves readability from the series using only half the signal.

### Perspectives

AI holds exciting prospects for nuclear medicine. Given how image quality is shaped by the relationship between injected activity and acquisition duration [26], our results raise several prospects.

First, a decrease in activity would limit the amount of irradiation of patients and staff. With an effective dose of 0.02 mSv per MBq [16], the average irradiation of patients in our cohort was 3.5 mSv, and so halving the activity would reduce the dose to 1.7 mSv. These activities remain very low and can even be considered negligible given the age of the patients and the cancer context.

The results of the VISION [27] study on ^177^Lu-PSMA internal radiation therapy have triggered a paradigm shift in prostate cancer treatment. This new and very promising therapy requires an imaging examination with PSMA before treatment initiation. In this context, demand for ^68^ Ga-PSMA PET scans is constantly increasing. AI is a good tool to help address this increasing demand for imaging. The germanium/gallium generators are scarce, which severely limits the availability of ^68^ Ga-PSMA, and so a decrease in dose per patient would allow a larger population to benefit from this technique. The current generation of gallium generators can prepare doses for 2–3 patients per day before dose reduction. From a technical point of view, it is possible to synthesize the radiopharmaceutical for twice as many patients by decreasing the doses, but a cost study would be needed to evaluate the feasibility of preparing 5–6 doses per day. However, given the radioactive decay during scanning time, it would increase but not double the number of doses with only one scanner available.

On the other hand, a decrease in acquisition times would serve to minimize motion artifacts and machine occupancy times while improving patient comfort. In addition, some studies [10, 21] have shown that the best delay after injection for the best contrast between tumor and non-tumor tissues is three hours. This three-hour imaging protocol for injections is, however, more restrictive in nuclear medicine departments, whereas most other tracers require a delay of only one hour. A decrease in acquisition time could thus facilitate the implementation of such a protocol.

Finally, the therapeutic impact of PSMA PET scans in this oligometastasis population is to guide stereotactic radiotherapy [18, 23, 28]. In this context, further studies are needed to evaluate the influence of SubtlePET® on fixation volumes.

### Limitations

First, the cohort included a small number of patients, making the localizations found according to PSA levels not similar to larger study [12]. Nevertheless, the increase of SUVmax in our cohort according to PSA level is consistent with the data from the literature [9].

Second, one of the biases of the study was that the junior reader only interpreted scans on Q.Clear-series reconstructions and not on VPFX-series reconstructions, as Q.Clear had been routinized in the department before his arrival. This explains why this reader qualitatively misclassified the VPFX series, which introduced a bias in the analysis of the overall quality and interpretability of the VPFX-derived series. Furthermore, PSMA-11 is not as specific as one might expect [11, 23, 29], and so this tracer comes with a learning curve. However, the three readers who participated in this study were familiar with interpreting these examinations in routine practice.

Third, for practical and especially ethical reasons, we do not have histological confirmation of lesions suspected of malignancy [30].

Fourth, this was a retrospective study that used the data available for reprocessing by SubtlePET®. Regarding routine feasibility, a prospective study [24] showed that the time lag between the end of the examination and the possibility of reading the SubtlePET® images was less than 10 min.

Fifth, we favored a subjective analysis of image quality over an objective analysis based on the use of computerized image comparison tools. Many studies [31–35] have used tools such as NRMSE (normalized root-mean-square error), PSNR (peak signal-to-noise ratio), or SSIM (structural similarity index), but all of these tools are tedious to set up and their clinical value remains limited [31].

Sixth, for data analysis, we did not use the PROMISE criteria [23] because at the time of the study, this classification was not currently used in the department.

Seventh, a bias of our study is the need of negative or inconclusive F-18-Choline PET/CT before giving access to 68-Ga-PSMA. This could explain why in our cohort, we found very small lesions.

Eighth, the choice of gold standard based on the referent was due to his experiment of reviewing almost all PSMA PET/CT during multi-disciplinary staff. For next studies, we shall use a consensus reading for gold standard.

Ninth, the height of patients were not available, so we could not do a subgroup analysis on BMI. Moreover, a study [36] showed that the BMI did not impact the liver SNR for Q.Clear. Also, no cross-validation was done between the two cameras but their CT reconstruction parameters were very similar.

Finally, the decrease in lesion SUVmax values makes it impossible to compare two successive scans if one of them is not processed with SubtlePET®. Moreover, as SubtlePET® decreases liver SBR, algorithm diagnostic value has to be evaluated in 177-Lu-PSMA population where patients are selected according metastasis vs liver uptake comparison [37]. In addition, almost all the lesions found were smaller than a centimeter and so were potentially influenced by the partial volume effect. This could explain the decrease in SUVmax values. However, a recent study [22] found the same results with a decrease of less than 10% in SUVmax value with ^18^FDG. The authors suggested that this decrease was mainly in small and moderate uptake. Further research would be needed to evaluate the biases introduced by this algorithm on lesions with much higher fixation intensities, such as in patients with high tumor burden.

## Conclusion

SubtlePET® maintains image quality and detectability of 68-Ga-PSMA PET using only half of the signal. Note, however, that the SubtlePET® algorithm significantly modifies the SUVmax values of the lesions and should therefore not be used for re-evaluation if the previous examination was reconstructed with a standard algorithm. This study shows that the SubtlePET®, which has already validated for ^18^FDG and amyloid PET, could also be used for ^68^ Ga-PSMA examinations.

## Data Availability

The datasets generated and/or analyzed over the course of this study are available from the corresponding author on reasonable request.

## Appendix 1: quantitative comparison of background measurement

**Table:**

Series	Mean SUV	Difference in SUV	CI 95% of the difference	Adjusted p	Bias
*QC4*					
Aorta background SUVmean	1.09				
Liver background SUVmean	5.07				
Gluteal background SUVmean	0.39				
Aorta background SUVmax	1.27				
Liver background SUVmax	5.83				
Gluteal background SUVmax	0.61				
*SQC1*					
Aorta background SUVmean	1.12	0.04	(− 0.02–0.09)	0.238	2.8%
Liver background SUVmean	5.27	0.22	(0.14–0.29)	*0.001*	3.9%
Gluteal background SUVmean	0.36	− 0.03	(− 0.04–0.01)	< *0.001*	− 7.7%
Aorta background SUVmax	1.37	0.10	(0.02–0.18)	*0.019*	7.8%
Liver background SUVmax	6.42	0.51	(0.37–0.66)	< *0.001*	10.1%
Gluteal background SUVmax	0.65	0.03	(− 0.03–0.8)	0.332	6.6%
*SQC2*					
Aorta background SUVmean	1.11	0.02	(0–0.05)	0.105	1.8%
Liver background SUVmean	5.25	0.21	(0.15–0.27)	*0.001*	3.6%
Gluteal background SUVmean	0.36	− 0.03	(− 0.04–0.01)	< *0.001*	− 7.7%
Aorta background SUVmax	1.27	0.00	(− 0.03–0.03)	0.883	0%
Liver background SUVmax	6.03	0.20	(0.12–0.26)	*0.001*	3.4%
Gluteal background SUVmax	0.53	− 0.08	(− 0.11–0.04)	< *0.001*	− 13.1%
*SQC3*					
Aorta background SUVmean	1.11	0.02	(− 0.01–0.04)	0.19	1.8%
Liver background SUVmean	5.26	0.23	(0.17–0.27)	*0.001*	3.7%
Gluteal background SUVmean	0.36	− 0.03	(− 0.04–0.02)	*0.001*	− 7.7%
Aorta background SUVmax	1.22	− 0.04	(− 0.08–0)	*0.04*	− 3.4%
Liver background SUVmax	5.93	0.10	(0.04–0.17)	*0.008*	1.7%
Gluteal background SUVmax	0.49	− 0.12	(− 0.16–0.09)	< *0.001*	− 19.6%
*SQC4*					
Aorta background SUVmean	1.09	0.01	(− 0.02–0.03)	0.527	0%
Liver background SUVmean	5.30	0.26	(0.2–0.33)	*0.001*	4.5%
Gluteal background SUVmean	0.35	− 0.03	(− 0.04–0.02)	< *0.001*	− 10.3%
Aorta background SUVmax	1.20	− 0.07	(− 0.1–0.04)	< *0.001*	− 5.5%
Liver background SUVmax	5.89	0.06	(0.01–0.12)	*0.04*	1%
Gluteal background SUVmax	0.48	− 0.13	(− 0.17–0.1)	< *0.001*	− 21.3%
*VP4*					
Aorta background SUVmean	1.04				
Liver background SUVmean	5.03				
Gluteal background SUVmean	0.39				
Aorta background SUVmax	1.34				
Liver background SUVmax	6.49				
Gluteal background SUVmax	0.69				
*SVP1*					
Aorta background SUVmean	1.11	0.07	(− 0.01–0.15)	0.115	6.7%
Liver background SUVmean	5.25	0.23	(0.15–0.31)	< *0.001*	4.4%
Gluteal background SUVmean	0.37	− 0.02	(− 0.04–0)	*0.04*	− 5.1%
Aorta background SUVmax	1.41	0.08	(− 0.05–0.19)	0.228	5.2%
Liver background SUVmax	7.11	0.64	(0.39–0.87)	< *0.001*	9.5%
Gluteal background SUVmax	0.66	− 0.03	(− 0.07–0.01)	0.228	− 4.3%
*SVP2*					
Aorta background SUVmean	1.10	0.06	(0.01–0.1)	*0.018*	5.8%
Liver background SUVmean	5.30	0.23	(0.16–0.29)	< *0.001*	5.3%
Gluteal background SUVmean	0.35	− 0.04	(− 0.05–0.02)	< *0.001*	− 10.3%
Aorta background SUVmax	1.33	− 0.01	(− 0.09–0.06)	0.777	− 0.7%
Liver background SUVmax	6.52	0.09	(0.07–0.21)	0.267	0.4%
Gluteal background SUVmax	0.55	− 0.14	(− 0.17–0.01)	< *0.001*	− 20.2%
*SVP3*					
Aorta background SUVmean	1.10	0.07	(0.03–0.1)	*0.006*	5.8%
Liver background SUVmean	5.25	0.25	(0.19–0.3)	< *0.001*	4.4%
Gluteal background SUVmean	0.35	− 0.04	(− 0.05–0.02)	< *0.001*	− 10.3%
Aorta background SUVmax	1.29	− 0.05	(− 0.11–0.01)	0.142	− 3.7%
Liver background SUVmax	6.28	− 0.6	(− 0.33–0)	0.069	− 3.2%
Gluteal background SUVmax	0.52	− 0.18	(− 0.22–0.13)	< *0.001*	− 24.6%
*SVP4*					
Aorta background SUVmean	1.07	0.03	(0–0.07)	0.095	2.9%
Liver background SUVmean	5.27	0.26	(0.21–0.32)	< *0.001*	4.8%
Gluteal background SUVmean	0.35	− 0.03	(− 0.04–0.02)	< *0.001*	− 10.3%
Aorta background SUVmax	1.22	− 0.11	(− 0.18–0.05)	*0.002*	− 8.9%
Liver background SUVmax	6.28	− 0.22	(− 0.3–0.13)	< *0.001*	− 3.2%
Gluteal background SUVmax	0.50	− 0.19	(− 0.22–0.16)	< *0.001*	− 27.5%

Difference = gold standard—study series; Bias = (study series—gold standard)/gold standard

## Appendix 2: difference in signal-to-background values as a function of patient weight

$${\text{SUV}}_{\max } \,{\text{lesion/SUVmean}}\,{\text{liver}}{\text{. Orange}}\,{ = }\,{\text{weight < median/Blue}}\,{ = }\,{\text{weight > median}}$$

## Appendix 3: difference in measured values as a function of camera

**Table:**

	Mean (SD)		Difference	Adjusted *p*	*p*
	PMT-based PET	SiPM-based PET			
*Aorta SUVmean*					
VP4	0.96 (0.16)	1.12 (0.22)	− 0.15	0.23	*0.037*
SVP1	1.06 (0.28)	1.16 (0.29)	− 0.1	0.798	0.359
SVP2	1.05 (0.16)	1.15 (0.2)	− 0.11	0.49	0.124
SVP3	1.07 (0.14)	1.14 (0.17)	− 0.08	0.558	0.179
SVP4	1.03 (0.12)	1.12 (0.15)	− 0.09	0.386	0.077
QC4	1.04 (0.15)	1.13 (0.18)	− 0.1	0.49	0.126
SQC1	1.03 (0.2)	1.21 (0.26)	− 0.18	0.23	*0.038*
SQC2	1.04 (0.12)	1.18 (0.18)	− 0.14	0.185	*0.024*
SQC3	1.06 (0.12)	1.15 (0.17)	− 0.1	0.386	0.082
SQC4	1.04 (0.12)	1.15 (0.16)	− 0.11	0.275	*0.049*
*Liver SUVmean*					
VP4	4.57 (1.42)	5.46 (1.79)	− 0.88	0.493	0.147
SVP1	4.82 (1.44)	5.69 (1.78)	− 0.87	0.493	0.152
SVP2	4.94 (1.76)	5.66 (1.86)	− 0.51	0.645	0.263
SVP3	4.81 (1.5)	5.69 (1.92)	− 0.61	0.581	0.202
SVP4	4.83 (1.52)	5.70 (1.91)	− 0.57	0.595	0.217
QC4	4.7 (1.37)	5.44 (1.77)	− 0.48	0.734	0.309
SQC1	4.87 (1.45)	5.67 (1.77)	− 0.8	0.571	0.188
SQC2	4.83 (1.47)	5.67 (1.86)	− 0.55	0.595	0.221
SQC3	4.82 (1.49)	5.70 (1.87)	− 0.88	0.526	0.165
SQC4	4.86 (1.49)	5.73 (1.92)	− 0.55	0.595	0.217
*Gluteal SUVmean*					
VP4	0.34 (0.05)	0.43 (0.09)	− 0.09	0.09	*0.003*
SVP1	0.32 (0.05)	0.42 (0.12)	− 0.1	0.09	*0.006*
SVP2	0.31 (0.05)	0.38 (0.09)	− 0.07	0.144	*0.017*
SVP3	0.32 (0.06)	0.39 (0.09)	− 0.07	0.125	*0.013*
SVP4	0.31 (0.05)	0.39 (0.08)	− 0.08	0.09	*0.005*
QC4	0.34 (0.04)	0.43 (0.08)	− 0.09	0.09	*0.001*
SQC1	0.31 (0.05)	0.40 (0.09)	− 0.09	0.09	*0.003*
SQC2	0.32 (0.05)	0.40 (0.09)	− 0.08	0.09	*0.006*
SQC3	0.32 (0.06)	0.39 (0.09)	− 0.07	0.144	*0.017*
SQC4	0.31 (0.05)	0.39 (0.09)	− 0.08	0.09	*0.006*
*Lesion SUVmax*					
VP4	6.74 (3.7)	8.36 (6.45)	− 0.4	0.99	0.896
SVP1	5.13 (3.32)	7.95 (7)	− 1.79	0.636	0.254
SVP2	5.62 (3.61)	7.72 (6.57)	− 1.19	0.926	0.508
SVP3	5.75 (3.04)	7.54 (6.4)	− 0.66	0.993	0.702
SVP4	5.49 (3.23)	7.64 (6.26)	− 0.87	0.946	0.602
QC4	8.94 (6.15)	8.89 (6.8)	− 0.09	0.99	0.99
SQC1	7.47 (5.28)	8.93 (7.69)	− 1.04	0.993	0.702
SQC2	7.03 (5.04)	7.92 (7.26)	− 0.49	0.99	0.771
SQC3	7.46 (4.97)	7.72 (7.04)	0.34	0.99	0.972
SQC4	7.26 (4.91)	7.52 (6.86)	0.38	0.99	0.917
*SBR = Lesion SUVmax/gluteal SUVmean*					
VP4	20.77 (10.9)	20.12 (16.23)	2.17	0.926	0.556
SVP1	16.99 (10.22)	20.47 (17.97)	− 0.86	0.99	0.771
SVP2	18.86 (11.84)	21.97 (19.44)	− 0.27	0.99	0.972
SVP3	19.18 (10.05)	21.91 (22.56)	0.31	0.99	0.862
SVP4	18.42 (10.44)	20.81 (18.34)	0.26	0.99	0.972
QC4	27 (18.38)	21.47 (16.91)	4.71	0.946	0.602
SQC1	24.64 (15.85)	23.41 (19.86)	2.1	0.946	0.602
SQC2	23.68 (17.09)	21.27 (19.79)	2.63	0.946	0.582
SQC3	24.67 (16.48)	22.02 (23.26)	3.17	0.89	0.464
SQC4	24.34 (16.67)	21.18 (22)	3.25	0.926	0.554

## Abbreviations

ALARA: As low as reasonably achievable
CI: Confidence interval
CNR: Contrast-to-noise ratio
^18^FDG: ^18^Fluoro-Desoxy-Glucose
MDT: Metastasis-directed therapy
NRMSE: Normalized root-mean-square error
OSEM: Ordered subset expectation maximization
PSA: Prostate-specific antigen
PSF: Point spread function
PSMA: Prostate-specific membrane antigen
PSNR: Peak signal-to-noise ratio
SBR: Signal-to-background ratio
SNR: Signal-to-noise ratio
SSIM: Structural similarity index
SUV: Standardized uptake value
PET: Positron emission tomography
TOF: Time of flight
TRT: Targeted radiotherapy
